# Clinical significance of psychotic-like experiences across U.S. ethnoracial groups

**DOI:** 10.1017/S0033291723001496

**Published:** 2023-12

**Authors:** Roberto Lewis-Fernández, Chih-nan Chen, Mark Olfson, Alejandro Interian, Margarita Alegría

**Affiliations:** 1 Department of Psychiatry, Columbia University and New York State Psychiatric Institute, New York, NY, USA; 2 Department of Economics, National Taipei University, Taipei, Taiwan, Republic of China; 3 Mental Health and Behavioral Sciences, VA New Jersey Healthcare System, Lyons, NJ, USA; 4 Disparities Research Unit, Department of Medicine, Massachusetts General Hospital/Harvard Medical School, Boston, MA, USA

**Keywords:** Clinical meaning of symptoms, cultural variation of psychiatric disorders, ethnoracial health disparities, psychotic-like experiences

## Abstract

**Background:**

Prevalence of *psychotic-like experiences* (PLEs) – reports of hallucinations and delusional thinking not meeting criteria for psychotic disorder – varies substantially across ethnoracial groups. What explains this range of PLE prevalence? Despite extensive research, the clinical significance of PLEs remains unclear. Are PLE prevalence and clinical severity differentially associated across ethnoracial groups?

**Methods:**

We examined the lifetime prevalence and clinical significance of PLEs across ethnoracial groups in the Collaborative Psychiatric Epidemiology Surveys (*N* = 11 139) using the Composite International Diagnostic Interview (CIDI) psychosis symptom screener. Outcomes included mental healthcare use (inpatient, outpatient), mental health morbidity (self-perceived poor/fair mental health, suicidal ideation or attempts), and impairment (role interference). Individuals with outcome onsets prior to PLE onset were excluded. We also examined associations of PLEs with CIDI diagnoses. Cox proportional-hazards regression and logistic regression modeling identified associations of interest.

**Results:**

Contrary to previous reports, only Asian Americans differed significantly from other U.S. ethnoracial groups, reporting lower lifetime prevalence (6.7% *v.* 8.0–11.9%) and mean number (0.09 *v.* 0.11–0.18) of PLEs. In multivariate analyses, PLE clinical significance showed limited ethnoracial variation among Asian Americans, non-Caribbean Latinos, and Afro-Caribbeans. In other groups, mental health outcomes showed significant ethnoracial clustering by outcome (e.g. hospitalization and role interference with Caribbean-Latino origin), possibly due to underlying differences in psychiatric disorder chronicity or treatment barriers.

**Conclusions:**

While there is limited ethnoracial variation in U.S. PLE prevalence, PLE clinical significance varies across U.S. ethnoracial groups. Clinicians should consider this variation when assessing PLEs to avoid exaggerating their clinical significance, contributing to mental healthcare disparities.

The prevalence of *psychotic-like experiences* (PLEs) – reports of hallucinations and delusional thinking not meeting criteria for psychotic disorder (Linscott & van Os, 2013) – varies by ethnoracial background and country of origin (Leaune et al., 2018; Nuevo et al., 2012; Oh, Yang, Anglin, & DeVylder, 2014). ‘Ethnoracial background’ denotes a person's identification as the intersection of two U.S. Census constructs – ‘race’ (e.g. Black, White) and ‘ethnicity’ (Latino, non-Latino). In nationally representative samples from 18 countries, lifetime prevalence of ≥1 PLEs was 1.2–14.9% and significantly higher in middle- and high-income countries than low-income ones (McGrath et al., 2015). In high-income countries, PLE elevations are most consistent among Black and/or Caribbean populations (Anglin et al., 2021; Johns, Nazroo, Bebbington, & Kuijpers, 2002; Leaune et al., 2018). In the USA, PLE prevalence is reported as significantly higher among Latinos and Blacks, and lower among Asian Americans, compared to non-Latino Whites (Cohen & Marino, 2013; DeVylder, Oh, Corcoran, & Lukens, 2014a).

What explains this broad range of PLE prevalence? Despite extensive research, the clinical significance of PLEs remains unclear (Powers, 2019). PLEs are considerably more common than psychotic disorders, with a median prevalence of 5–9% among adults and youth (Kelleher et al., 2012; Linscott & van Os, 2013; McGrath et al., 2015), and are often transient and non-distressing (Hanssen, Bak, Bijl, Vollebergh, & van Os, 2005; McGrath et al., 2015). Only 7% of individuals with PLEs develop a psychotic disorder (Linscott & van Os, 2013). Still, the relative risk of conversion rises in a dose–response relationship with PLE severity (Kaymaz et al., 2012) and PLEs share many risk factors with schizophrenia (Kelleher & Cannon, 2011). However, PLEs also predict non-psychotic common mental disorders (CMDs) (Kaymaz et al., 2012) and people with PLEs share genetic liability with a broad range of mental disorders (Legge et al., 2019).

At the population level, does the clinical significance of PLEs vary across ethnoracial groups, complicating their etiological and predictive meaning? For example, are PLEs more prevalent in ethnoracial groups that consider them minimally impairing, familiar cultural idioms of distress (Johns et al., 2002; Lewis-Fernández et al., 2009)? Or is the clinical significance of PLEs equivalent across ethnoracial groups, making PLE prevalence variation a reliable indicator of cross-ethnoracial/cultural variation in psychopathology?

PLEs are associated with mental health morbidity, impairment, treatment-seeking, and mental disorder severity and comorbidity (DeVylder, Burnette, & Yang, 2014b), suicidal ideation and behaviors (Honings, Drukker, Groen, & van Os, 2016), and impaired social functioning (Oh, Koyanagi, Kelleher, & DeVylder, 2018).

When examined by ethnoracial background, the clinical significance of PLEs displays a complex pattern. Partly, this is because ethnoracial groupings are social proxies for structural conditions, cultural norms, and contextual factors that may contribute specific risks for PLEs. Although the few relevant studies emphasize ethnoracial similarities in clinical significance (e.g. DeVylder et al., 2014b; King et al., 2005), unexplained differences often remain across ethnoracial groups in the number and strength of clinical associations (DeVylder et al., 2014b; Oh et al., 2018; Vanheusden et al., 2008). Several limitations have hindered ethnoracial comparisons in the strength of association between PLEs and adverse outcomes: ignoring the timing of PLE and morbidity outcome onsets, confounding their temporal association (e.g. DeVylder et al., 2014b); examining few adverse outcomes (e.g. King et al., 2005); analyzing small samples (e.g. Vanheusden et al., 2008); and using country, not ethnoracial background, as unit of analysis (e.g. Nuevo et al., 2012). The range of inter-ethnoracial differences is often unexamined, usually because studies collapsed ethnoracial groups – e.g. Caribbean and non-Caribbean-origin populations (e.g. DeVylder et al., 2014b) or Asian Americans and Latinos (e.g. Oh et al., 2018) – or only compared to non-Latino Whites (e.g. Vanheusden et al., 2008).

This study examines whether the social-identity construct of ethnoracial background is associated with differences in prevalence and clinical significance of PLEs. Clinical significance was assessed as mental healthcare use (inpatient, outpatient), morbidity (self-perceived poor/fair mental health, suicidal ideation or attempts), and impairment (role interference). We also assessed whether respondents with lifetime PLEs had higher lifetime prevalence of CMDs. *A priori*, we hypothesized that ethnoracial groups with lower PLE prevalence would report stronger correlations of PLEs with mental health morbidity and impairment. Such an inverse correlation would suggest that ethnoracial groups with stronger associations of PLEs with psychopathology may be less likely to report PLEs without substantial impairment.

In contrast, groups that express PLEs within a broader range of situations, including subclinical distress and spiritual experiences, may more readily report them. Estimating ethnoracial variation in PLE clinical significance may clarify their wide prevalence range and suggest structural-contextual factors underlying variation by ethnoracial background. It may also enhance the cross-ethnoracial reliability of clinicians' use of PLEs as severity markers of psychopathology (Kelleher & Cannon, 2011).

## Methods

### Collaborative psychiatric epidemiological surveys (CPES)

Three national surveys of the non-institutionalized adult U.S. population comprise the CPES: the National Comorbidity Survey Replication (NCS-R; Kessler & Merikangas, 2004), the National Survey of American Life (NSAL; Jackson et al., 2004), and the National Latino and Asian American Study (NLAAS; Alegría et al., 2004); similar methods allow for their combination into a single, nationally representative dataset (Heeringa et al., 2004). Only English-speaking respondents participated in the NCS-R and NSAL; the NLAAS included participants who spoke English, Spanish, or one of four Asian languages (Alegría et al., 2004). Interviewers were matched to participants by racialized (NSAL) or linguistic/cultural (NLAAS) background.

Our sample includes CPES respondents who completed the World Mental Health-Composite International Diagnostic Interview (WMH-CIDI) psychosis-symptom screener: NSAL African Americans (AfrAms; *N* = 3414) and Black Americans of Afro-Caribbean origin (AfrCaribs; *N* = 1400); NLAAS Asian Americans (AsAms; *N* = 2092), Caribbean Latinos (CaribLats; *N* = 1152), and non-Caribbean Latinos (NCLats; *n* = 1399; 68.7% of Mexican origin); and a random subsample of the NCS-R non-Latino Whites (NLWs; *N* = 1705) who completed the WMH-CIDI ‘long-form’. The Internal Review Boards of the principal investigators' institutions approved study methods and protocols; all participants provided informed consent.

### Measures

Endorsement and age of onset of self-reported PLEs were assessed using the WMH-CIDI psychosis-symptom screener (Kessler & Üstün, 2004), which assesses visual hallucinations, auditory hallucinations, thought insertion/withdrawal, delusions of control, delusions of reference, and persecutory delusions. Only experiences that occurred when not ‘dreaming or half-asleep or under the influence of alcohol or drugs’ were included. Respondents were classified as having PLEs if they endorsed ≥1 PLEs over their lifetime. Because of ethnoracial variation in access to clinical services and in misdiagnosis of mood conditions as psychotic disorders (Gara et al., 2012), we did not exclude individuals with a clinical diagnosis of psychotic disorder. Excluding such individuals would potentially exert a larger confounding effect across ethnoracial groups than including them.

Six measures obtained in all CPES samples comprised the mental health outcomes: ever having an overnight hospital admission for problems with emotions, nerves, mental health, or use of alcohol or drugs (psychiatric hospitalization); ever having an outpatient contact with specialty-mental-health or general-health professionals for mental health problems (outpatient mental healthcare); current self-perceived mental health (fair/poor *v.* excellent/very good/good); ever having self-reported suicidal ideation or attempts (yes/no for each, asked separately); and role interference, assessed as inability to fulfill social roles due to mental health problems on any day over the last month (*v.* no days) (Rehm et al., 1999).

Socio-demographic correlates included age at interview (18–34, 35–49, ≥50 years), gender (male/female), education (0–11, 12, 13–15, ≥16 years), annual household income (<$15 000, $15 000–34 999, $35 000–74 999, ≥$75 000), and marital status (married/cohabiting *v.* never married/separated/widowed/divorced). WMH-CIDI lifetime DSM-IV psychiatric diagnoses were grouped as any depressive disorder (major depressive episode, dysthymia), any anxiety or trauma-related disorder (agoraphobia without panic disorder, panic disorder, generalized anxiety disorder, social phobia, posttraumatic stress disorder), any substance use disorder (alcohol abuse/dependence, drug abuse/dependence), and any mental disorder (any of the 11 diagnoses). The sum of individual disorders yielded the number of lifetime disorders per respondent.

### Statistical analysis

Categorical variables were compared with the Rao–Scott statistic (Rao & Scott, 1984) and continuous variables using adjusted Wald tests. Tests for outliers and multicollinearity were negative. Respondents with missing data on PLE items were excluded from the analytic sample. All analyses were conducted applying sampling weights (Heeringa et al., 2004) to generalize results to the U.S. population. For NLW respondents, we utilized the weights for the NCS-R sample administered additional measures (‘long-form’), including a random subsample completing the psychosis symptom screener; the ‘long-form’ survey oversampled NCS-R respondents with psychopathology plus a probability subsample of others (Kessler et al., 2005). Cross-tabulations display age-and-gender-adjusted ethnoracial differences in lifetime endorsement of PLEs for the full sample (*N* = 11 139). Significant omnibus tests (*p* < 0.05) were followed by *post-hoc* pairwise comparisons across individual ethnoracial groups, applying Benjamini–Hochberg correction for multiple comparisons (Thissen, Steinberg, & Kuang, 2002). Among respondents with lifetime PLEs (*n* = 1138), the distribution of lifetime CMDs and mental health outcomes after PLE onset were compared across ethnoracial groups. Supplementary analyses examined ethnoracial variation of specific PLEs, other PLE-related characteristics, and socio-demographic correlates (online Supplementary Tables S1 and S2).

In the sample with lifetime PLEs, NLWs were compared to other ethnoracial groups using Cox proportional-hazards regression models to examine the strength of association between PLEs and four lifetime mental health outcomes after PLE onset (psychiatric hospitalization, outpatient mental healthcare, suicidal ideation, suicide attempts). Survival models, which include time as a covariate, tend to outperform logistic models that ignore time variation (Ngwa et al., 2016). *Post-hoc* pairwise comparisons were limited to those in which the hazards estimates from one group's full model fell outside the comparator group's confidence interval (CI) range. We excluded participants who first experienced an outcome before PLE onset by comparing the date of first onset of each outcome to the date of first PLE onset. Possible explanatory variables included age at PLE onset, gender, education, household income, marital status, and number of lifetime CMDs. In sensitivity analyses, we removed the number of CMDs in backward regressions to test its role in multivariate models. Given the marked variation of nativity status across groups, we included it as a potential explanatory variable in separate supplementary analyses of the PLE-positive sample, combining AfrAms and AfrCaribs into one group and CaribLats and NCLats into another group due to sample size limitations (online Supplementary Table S3).

In the full sample (*N* = 11 139), we also examined associations between lifetime PLEs (*n* = 1138) and two outcomes current at time of interview – current self-perceived mental health status and role interference in the last month – using separate logistic regressions for each ethnoracial group, adjusting for PLE duration and other explanatory variables. The interaction between ethnoracial background and PLE status on each outcome was assessed separately, with NLWs as the reference group. *Post-hoc* pairwise comparisons of other ethnoracial groups and nativity analyses were conducted as above.

STATA 15.1 Survey Analysis procedures accounted for the complex sample design (Stata Corp, 2017).

## Results

### Lifetime PLE prevalence

Overall lifetime PLE endorsement was 9.1%, with significant variation by ethnoracial background (omnibus *p* < 0.0001) driven mainly by lower PLE prevalence among AsAm respondents (Table 1). NLWs (8.0%), CaribLats (10.8%), and AfrAms (11.9%) were significantly more likely than AsAms (6.7%) to report lifetime PLEs, after adjusting for multiple comparisons. The mean (s.d.) number of lifetime PLEs showed the same endorsement pattern (Table 1). The prevalence of visual hallucinations ranged from 4.6% (AsAms) to 9.5% (AfrCaribs) (omnibus *p* < 0.0001) and was significantly higher in AfrAms than NLWs and AsAms. Auditory hallucinations ranged from 2.5% (AfrCaribs) to 6.2% (CaribLats) (omnibus *p* < 0.0001); they were significantly more frequent among AfrAms and CaribLats than NLWs, AsAms, or AfrCaribs and among NCLats than AsAms. After adjustment for multiple comparisons, delusional thinking did not show significant ethnoracial variation.

**Table 1.** Lifetime PLEs among CPES respondents, adjusted by age and gender (*N* = 11 139)

		Total	NCS-R non-Latino Whites (I)	NLAAS Caribbean Latinos (II)	NLAAS non-Caribbean Latinos (III)	NLAAS Asian Americans (IV)	NSAL African Americans (V)	NSAL Afro-Caribbeans (VI)	Omnibus test	Benjamini– Hochberg *post-hoc* comparison
		(*N* = 11 139)	(*n* = 1682)^a,b^	(*n* = 1152)^b,c^	(*n* = 1399)^b,d^	(*n* = 2092)^b^	(*n* = 3414)^b^	(*n* = 1400)^b,e^		
Any PLEs	Mean	9.1%	8.0%	10.8%	9.6%	6.7%	11.9%	10.6%	<0.0001	I, II, V > IV
	s.e.	0.4%	0.7%	1.0%	1.0%	0.6%	0.9%	1.8%		
Number of PLEs	Mean	0.13	0.11	0.18	0.13	0.09	0.16	0.16	<0.0001	I, II, V > IV
	s.e.	0.01	0.01	0.02	0.01	0.01	0.01	0.02		
Visual hallucinations	Mean	6.3%	5.4%	7.2%	6.2%	4.6%	8.7%	9.5%	<0.0001	V > I, IV
	s.e.	0.4%	0.7%	0.8%	0.6%	0.5%	0.7%	1.8%		
Auditory hallucinations	Mean	3.9%	3.2%	6.2%	4.7%	2.6%	5.3%	2.5%	<0.0001	II, V > I II, III, V > IV II, V > VI
	s.e.	0.3%	0.4%	0.9%	0.6%	0.4%	0.6%	0.6%		
Thought insertion/withdrawal	Mean	0.4%	0.5%	1.3%	0.3%	0.4%	0.3%	0.8%	0.26	
	s.e.	0.1%	0.1%	0.4%	0.2%	0.2%	0.1%	0.4%		
Delusions of control	Mean	0.2%	0.1%	0.9%	0.2%	0.2%	0.3%	0.6%	0.21	
	s.e.	0.1%	0.1%	0.4%	0.1%	0.1%	0.1%	0.4%		
Delusions of reference	Mean	0.7%	0.7%	1.3%	0.9%	0.6%	0.6%	0.8%	0.68	
	s.e.	0.1%	0.2%	0.5%	0.3%	0.2%	0.1%	0.4%		
Persecutory delusions	Mean	1.0%	0.9%	1.3%	1.0%	0.5%	1.2%	2.1%	0.11	
	s.e.	0.1%	0.2%	0.5%	0.5%	0.2%	0.3%	1.1%		

s.e., standard error.

^a^Only the NCS-R random subsample that completed the psychosis symptom screener was included.

^b^Respondents with missing PLE data were dropped from the sample.

^c^Caribbean Latinos include respondents who identify as Puerto Rican, Cuban, or Dominican.

^d^Non-Caribbean Latinos include all other respondents who identify as Latinos (68.7% of Mexican origin).

^e^Afro-Caribbean respondents identified as Black and reported West Indian or Caribbean descent in their own, their parents', or previous generations.

*Note*: All analyses were conducted applying sampling weights to generalize results to the U.S. population.

In the PLE-positive group, AfrCaribs were significantly younger at PLE onset than NLWs, CaribLats, or AfrAms (online Supplementary Table S1). PLE frequency varied significantly by ethnoracial background in omnibus tests, but not after Benjamini–Hochberg correction (online Supplementary Table S1). Significant socio-demographic variation among respondents with PLEs followed population-level ethnoracial differences in the CPES sample (online Supplementary Table S2; Alegria, Woo, Takeuchi, & Jackson, 2009), suggesting the PLE-group variation resulted from overall population differences.

### Bivariate mental health outcomes by ethnoracial background

#### Lifetime prevalence of CMDs

In analyses of the age-and-gender-adjusted sample with PLEs (*n* = 1138), the prevalence of most lifetime CMDs varied significantly by ethnoracial background (omnibus *p*'s < 0.02–0.001) (Table 2). NLWs had the highest lifetime prevalence of any CMD, including separately for depression and anxiety, and the largest proportion of respondents with multiple mental disorders. By contrast, AsAms had the lowest lifetime prevalence and proportion of respondents with multiple disorders.

**Table 2.** Lifetime psychiatric disorders and mental health outcomes of CPES respondents with lifetime PLEs, adjusted by age and gender (*N* = 1138)

Number of lifetime PLE endorsers	Total (%)	NCS-R non-Latino Whites (I) (%)	NLAAS Caribbean Latinos (II) (%)	NLAAS non-Caribbean Latinos (III) (%)	NLAAS Asian-Americans (IV) (%)	NSAL African-Americans (V) (%)	NSAL Afro-Caribbeans (VI) (%)	Omnibus test	Benjamini– Hochberg *post-hoc* comparison
	(*n* = 1138)	(*n* = 181)^a,b^	(*n* = 143)^b,c^	(*n* = 136)^b,d^	(*n* = 121)^b^	(*n* = 427)^b^	(*n* = 130)^b,e^	*p* value	
*Lifetime psychiatric disorder*									
Any lifetime mental disorder	56.7	63.4	58.4	53.2	32.8	52.0	55.4	0.0184	I, II, III, V > IV
Any lifetime depressive disorder	30.5	38.4	26.7	25.9	17.3	23.2	28.8	0.0017	I > IV
Any lifetime anxiety disorder	39.0	46.6	39.4	27.2	22.7	36.6	34.1	0.0004	I > III, IV
Any lifetime substance use disorder	23.5	27.5	22.7	24.3	10.7	18.8	22.4	0.24	
Number of lifetime disorders									
0	43.3	36.6	41.6	46.8	67.2	48.0	44.6	0.0116	I *v.* IV
1	18.5	18.5	19.8	16.0	13.9	20.2	25.9		
2	20.4	20.6	23.3	24.8	14.7	18.3	13.6		
3+	17.9	24.2	15.4	12.4	4.3	13.6	15.8		
*Mental health outcomes*									
Psychiatric hospitalization after PLE onset	12.3	14.8	20.4	6.1	6.8	10.7	24.9	0.0006	II > III, IV
Outpatient care after PLE onset	34.0	39.3	37.0	21.2	21.7	34.6	40.0	0.0019	I, V > III
Suicidal ideation after PLE onset	20.0	25.4	19.7	15.1	8.4	16.1	17.1	0.0140	I > IV
Suicide attempt after PLE onset	9.7	10.9	8.9	10.9	5.1	7.4	21.4	0.34	
Fair or poor mental health currently	19.3	20.7	21.1	15.8	12.0	20.6	31.9	0.07	
Any days out of role in last 30 days	10.1	10.0	10.1	4.6	2.3	15.0	19.8	<0.0001	V > III, IV

PLE, psychotic-like experience.

^a^Only the NCS-R random subsample that completed the psychosis symptom screener was included.

^b^Respondents with missing PLE data were dropped from the sample.

^c^Caribbean Latinos include respondents who identify as Puerto Rican, Cuban, or Dominican.

^d^Non-Caribbean Latinos include all other respondents who identify as Latinos (68.7% of Mexican origin).

^e^Afro-Caribbean respondents identified as Black and reported West Indian or Caribbean descent in their own, their parents', or previous generations.

#### Lifetime prevalence of mental health outcomes after PLE onset

Several lifetime mental health outcomes also varied significantly across ethnoracial groups (omnibus *p*'s < 0.015–0.0001) (Table 2). After Benjamini–Hochberg adjustment, psychiatric hospitalization after PLE onset was significantly higher among CaribLats than NCLats or AsAms. Outpatient mental healthcare after PLE onset was significantly more common among NLWs and AfrAms than NCLats. NLWs were significantly more likely than AsAms to report suicidal ideation following the onset of PLEs. There were no statistically significant ethnoracial differences in the prevalence of suicide attempts after PLE onset.

#### Current mental health outcomes

The sample with lifetime PLEs showed no significant ethnoracial variation in fair/poor self-rated mental health at the interview. After Benjamini–Hochberg correction, AfrAms had significantly higher role interference than NCLats and AsAms.

### Multivariate analyses of mental health outcomes by ethnoracial background

#### Lifetime mental health outcomes

Among individuals with lifetime PLEs without hospitalizations before PLE onset, the adjusted hazards of first psychiatric hospitalization following PLE onset were not significantly different for any minoritized ethnoracial group compared to NLWs (Table 3). Four *post-hoc* pairwise comparisons of hazard ratios (HRs) among other ethnoracial groups were selected by inspection (see ‘Methods’). Lower hazards of hospitalization after PLE onset were found for NCLats (HR = 0.45; 95% CI 0.21–0.98) and AfrAms (HR = 0.52; 95% CI 0.30–0.92) compared to CaribLats.

**Table 3.** HRs of lifetime mental health outcomes by lifetime PLEs, adjusted by socio-demographic and clinical variables in CPES (*N*s = 917–1074)

	NCS-R non-Latino Whites^a,b^		NLAAS Caribbean Latinos^b,c^			NLAAS non-Caribbean Latinos^b,d^			NLAAS Asian Americans^b^			NSAL African Americans^b^			NSAL Afro-Caribbeans^b,e^			
	HR	95% CI	HR	95% CI		HR	95% CI		HR	95% CI		HR	95% CI		HR		95% CI	
	**(*n* = 168)**		**(*n* = 135)**			**(*n* = 127)**			**(*n* = 118)**			**(*n* = 402)**			**(*n* = 124)**			
**Psychiatric hospitalization after PLE onset (*n* = 1074)**	1		1.21	0.59	2.47	0.56	0.26	1.18	0.57	0.25	1.29	0.64	0.37	1.09		1.33	0.52	3.41
	**(*n* = 133)**		**(*n* = 117)**			**(*n* = 103)**			**(*n* = 108)**			**(*n* = 346)**			**(*n* = 110)**			
**Outpatient mental health care after PLE onset (*n* = 917)**	1		0.80	0.48	1.36	0.62	0.36	1.20	0.60	0.30	1.20	**0.65**	**0.43**	**0.99**	**	0.74	0.34	1.61
	**(*n* = 157)**		**(*n* = 128)**			**(*n* = 121)**			**(*n* = 110)**			**(*n* = 380)**			**(*n* = 119)**			
**Suicidal ideation after PLE onset (*n* = 1015)**	1		0.56	0.28	1.11	0.75	0.40	1.39	0.49	0.19	1.26	0.58	0.33	1.01		0.84	0.28	2.49
	**(*n* = 167)**		**(*n* = 127)**			**(*n* = 122)**			**(*n* = 116)**			**(*n* = 396)**			**(*n* = 120)**			
**Suicide attempt after PLE onset (*n* = 1048)**	1		0.53	0.23	1.24	1.10	0.39	3.12	1.46	0.69	3.09	0.50	0.20	1.22		1.91	0.34	10.92

PLE, psychotic-like experience.

^a^Only the NCS-R random subsample that completed the psychosis symptom screener was included.

^b^Respondents with missing PLE data were dropped from the sample.

^c^Caribbean Latinos include respondents who identify as Puerto Rican, Cuban, or Dominican.

^d^Non-Caribbean Latinos include all other respondents who identify as Latinos (68.7% of Mexican origin).

^e^Afro-Caribbean respondents identified as Black and reported West Indian or Caribbean descent in their own, their parents', or previous generations.

*Note*: Bolded values are statistically significant: **p < 0.01. Cox regressions adjusting for age of PLE onset, education, household annual income, marital status, and number of lifetime psychiatric disorders.

Significantly lower hazards of outpatient mental healthcare associated with PLEs were observed relative to NLWs among AfrAms (HR = 0.65; 95% CI 0.43–0.99) who had not received outpatient care before their PLE onset. Two *post-hoc* pairwise comparisons of HRs among other ethnoracial groups were selected by inspection; neither was statistically significant.

The minoritized ethnoracial groups did not differ from NLWs in their hazards of suicidal ideation or attempts after PLE onset. *Post-hoc* pairwise comparisons of HRs selected by inspection for suicidal ideation or attempts (one each) among other ethnoracial groups also yielded non-significant differences.

#### Current mental health outcomes

After controlling for background socio-demographic characteristics, number of lifetime CMDs, and duration of PLEs in the full sample (*N* = 11 139), lifetime PLEs were significantly associated with current poor/fair mental health among NLWs [odds ratio (OR) = 5.09; 95% CI 1.26–20.50] and AfrAms (OR = 1.98; 95% CI 1.20–3.24) (Table 4). As compared to NLWs, however, there were no significant pairwise ethnoracial differences in the strength of association between lifetime PLEs and current poor/fair mental health.

**Table 4.** Comparative odds of current mental health outcomes by lifetime PLEs across ethnoracial groups in the CPES (*N* = 11 139)

Total CPES sample = 11 139	NCS-R non-Latino Whites				NLAAS Caribbean Latinos			NLAAS non-Caribbean Latinos			NLAAS Asian-Americans			NSAL African- Americans				NSAL Afro-Caribbeans		
	(*n* = 1682)^a,b^				(*n* = 1152)^b,c^			(*n* = 1399)^b,d^			(*n* = 2092)^b^			(*n* = 3414)^b^				(*n* = 1400)^b,e^		
**Number of PLE endorsers**		**181**				**143**			**136**			**121**			**427**				**130**	
	OR	95% CI			OR	95% CI		OR	95% CI		OR	95% CI		OR	95% CI			OR	95% CI	
**Fair or poor mental health currently**	**5.09**	**1.26**	**20.50**	*	1.89	0.66	5.45	2.07	0.82	5.22	1.43	0.68	3.00	**1.98**	**1.20**	**3.24**	**	1.33	0.75	2.33
***p* values of interaction terms**^f^	Reference				0.32			0.21			0.99			0.53				0.31		
**Days out of role in last 30 days**	**3.72**	**1.57**	**8.85**	**	2.75	0.87	8.67	2.06	0.92	4.60	4.17	0.42	40.9	2.01	0.97	4.18		0.63	0.18	2.19
***p* values of interaction terms**^g^	Reference				0.99			0.06			0.68			**0.02**			*	0.07		

PLE: psychotic-like experience.

^a^Only the NCS-R random subsample that completed the psychosis symptom screener was included.

^b^Respondents with missing PLE data were dropped from the sample.

^c^Caribbean Latinos include respondents who identify as Puerto Rican, Cuban, or Dominican.

^d^Non-Caribbean Latinos include all other respondents who identify as Latinos (68.7% of Mexican origin).

^e^Afro-Caribbean respondents identified as Black and reported West Indian or Caribbean descent in their own, their parents', or previous generations.

^f^*p* values are for the interaction between ethnoracial group membership (non-Latino Whites, reference) and lifetime PLEs with current fair or poor mental health.

^g^*p* values are for the interaction between ethnoracial group membership (non-Latino Whites, reference) and lifetime PLEs with days out of role in the last 30 days.

*Note*: Bolded values are statistically significant: *p < 0.05; **p < 0.01. Logistic regressions adjusting for age of PLE onset, education, household annual income, marital status, number of lifetime psychiatric disorders, and duration of PLEs.

In the fully adjusted model, PLEs were significantly associated with role interference for NLWs (OR = 3.72; 95% CI 1.57–8.85). A significant interaction between PLEs and higher odds of role interference was found for AfrAms relative to NLWs (*p* = 0.02). Of the four *post-hoc* pairwise comparisons examined among other ethnoracial groups, the only significant comparison was lower odds of role interference among AfrCaribs compared to CaribLats (*p* = 0.02).

### Study hypothesis

Table 5 summarizes the significant bivariate and multivariate findings on ethnoracial variation in clinical outcomes associated with PLEs and juxtaposes them to PLE prevalences. These results contradict our hypothesis of an inverse correlation across ethnoracial groups between PLE prevalence and clinical significance: AsAms, for example, had both the lowest PLE prevalence and the lowest PLE clinical significance.

**Table 5.** Prevalence of PLEs and PLE-related functional outcomes across ethnoracial groups

Ethnoracial group	% PLE	Bivariate analyses	Multivariate analyses
Asian American (AsAm)	6.7		
Non-Latino White (NLW)	8.0	>NCLat (outpatient care)	>AfrAm (outpatient care)
		>AsAm (suicidal ideation)	
Non-Caribbean Latino (NCLat)	9.6		
Afro-Caribbean (AfrCarib)	10.6		
Caribbean Latino (CaribLat)	10.8	>NCLat (hospitalization)	>NCLat (hospitalization)
		>AsAm (hospitalization)	>AfrAm (hospitalization)
			>AfrCarib (role interference)
African American (AfrAm)	11.9	>NCLat (outpatient care, role interference)	>NLW (role interference)
		>AsAm (role interference)	

PLE, psychotic-like experience.

*Note*: ‘>’ indicates that PLEs experienced by the ethnoracial group in the first column are associated with significantly greater clinical and functional interference than PLEs experienced by the ethnoracial groups following the ‘>’ sign.

## Discussion

We examined the prevalence and clinical significance of self-reported PLEs in a representative U.S. sample, assessing variations across the social construct of ethnoracial background. Although the CPES was fielded in the early 2000s, it remains the best data source on PLEs in U.S. adults, since it includes a weighted nationally representative sample with sufficient ethnoracial diversity, uses the same PLE and mental health outcome measures across sub-studies, and includes ethnically or linguistically matched assessments.

While international epidemiological research reveals higher PLE prevalence among Black or Caribbean-descent individuals and lower prevalence among those of Asian origin, these findings were only partly confirmed. AsAms had significantly lower PLE prevalence and number of PLEs than NLWs, CaribLats, and AfrAms and were less likely to report auditory or visual hallucinations than several ethnoracial groups. But prevalence according to Black or Caribbean descent was inconsistent: AfrAms were significantly more likely to report auditory and visual hallucinations than NLWs but also more so than AfrCaribs, who did not differ statistically from NLWs. We observed a similar mixed pattern for auditory hallucinations for Caribbean origin for CaribLats, AfrCaribs, and NLWs; and the age of PLE onset showed significant intra-Black, intra-Caribbean variation.

The clinical significance of PLEs also showed ethnoracial variation. In bivariate analyses, AsAms with PLE's had lower risk of mental healthcare use, morbidity, and impairment after PLE onset than other groups; PLEs in nearly all ethnoracial groups were more likely to be associated with CMDs than AsAms. Among non-AsAm respondents with PLEs, the type of mental health outcome clustered by ethnoracial group. Relative to other groups with PLEs: NLWs with PLEs were significantly more likely to report CMDs, suicidal ideation, and outpatient treatment; AfrAms with PLEs reported more role interference and outpatient care; and CaribLats with PLEs, more hospitalization and role interference. Covariate adjustment eliminated significance differences relative to AsAms but not across several other groups. NCLats and AfrCaribs with PLEs were not associated with any specific type of mental health outcome.

### Ethnoracial variation in PLE prevalence

Our prevalence findings show similarities and differences with previous research. Mean U.S. lifetime PLE prevalence (9.1%) is within the international range (1.2–14.9%), though higher than the cross-national median lifetime prevalence estimates among adults of 5–6% in meta-analyses (Linscott & van Os, 2013; McGrath et al., 2015). However, we found less ethnoracial variation than most studies, which reveal lower prevalence among Asian-origin respondents and higher PLE prevalence among African- or Caribbean-descent individuals (DeVylder et al., 2014a; Johns et al., 2002; Leaune et al., 2018). Possible reasons for this discrepancy include differences in sampling, weighting, ethnoracial-group inclusion, prevalence period, PLE measurement, language of assessment, covariate selection, and analytical scheme (e.g. we included respondents with clinical psychotic-disorder diagnoses). Some prevalence reports have also raised methodological critiques (DeVylder, 2014).

The lower prevalence of PLEs among AsAms than other groups confirms past reports from the CPES (DeVylder et al., 2014a; Oh et al., 2014) and is consistent with low community rates of CMDs in U.S. Asian-origin populations (Xu et al., 2011). Potential explanations for both findings include higher proportions of foreign-born respondents than in other ethnoracial groups, who tend to report lower prevalence of mental health problems (Breslau & Chang, 2006), possibly related to lower duration of exposure to U.S.-based racial discrimination than native-born individuals (Gee, Spencer, Chen, Yip, & Takeuchi, 2007). However, including nativity in the model (online Supplementary Table S3) did not alter our findings. Other explanations include lower rates of substance use disorders, driving down risk for other psychiatric disorders (Xu et al., 2011); response bias due to higher stigmatization of mental illness among AsAms (Gee et al., 2007); or culture-specific expressions of emotional distress, leading to under-reporting (Breslau & Chang, 2006).

### Ethnoracial variation in clinical significance of PLEs

Variation in PLE clinical significance was notable among AsAms in bivariate analyses. This variation was attenuated after covariate adjustment, suggesting that demographic or clinical correlates account for a portion of the variance in PLE clinical significance among AsAms, relative to other ethnoracial groups. In backward regression analyses, removing the CMD covariate revealed significant differences between AsAms and other groups for suicidal ideation and hospitalization and a trend-level finding (*p* = 0.056) for role interference that persisted after demographic adjustments, suggesting that PLEs in AsAms are closely associated with CMDs.

Among non-AsAms with PLEs, ethnoracial background was associated either with non-significant variation in clinical significance (NCLats, AfrCaribs) or with a specific type of outcome (NLWs, CaribLats, AfrAms). Multivariate modeling suggests this variation was not due solely to demographic or clinical differences. The ethnoracial labels may indicate underlying socio-structural characteristics that pattern clinical significance outcomes. The association between NLWs and outpatient care (*v.* NCLats and AfrAms) may be due to longstanding disparities in access to ambulatory mental-health services among minoritized groups relative to NLWs (Chow, Jaffee, & Snowden, 2003). The fact that this association persisted in multivariate analyses suggests that, in NLWs, PLEs may be associated with using outpatient care independent of other covariates. The associations between AfrAms and role interference and between CaribLats, hospitalization, and role interference in multivariate models suggest that PLEs may be connected to these groups' greater chronicity (Vilsaint et al., 2019; Williams et al., 2007) and involuntary inpatient admissions (Bhalla et al., 2022).

But why do NCLats and AfrCaribs show no specific association relative to other ethnoracial groups between PLEs and mental health outcomes in bivariate or multivariate analyses? These groups also face delays in seeking mental health care (Wells, Klap, Koike, & Sherbourne, 2001), often reporting persistent and severe mental disorders (Williams et al., 2007). In addition to socio-structural barriers, ethnoracial labels can signal cultural variation in the association of individual symptoms with adverse outcomes. Distinct cultural traditions of meaning and social practice may be impacting the acceptability and functional impact of PLEs (Kirmayer, Gómez-Carrillo, & Veissière, 2017) by influencing differential concern, social supports, stigmatization, and help-seeking processes (Kaiser et al., 2015; Lewis-Fernández & Kirmayer, 2019). The anthropological term *idiom of distress* indicates the function of a symptom as an expression of suffering intended to communicate a specific message (Nichter, 2010). Superficially similar symptoms may signal distinct messages – including marital discord, social-status dissatisfaction, demoralization, or severe psychopathology – depending on the meaning constellations encoded in cultural traditions and the circumstances of the individual sufferer. For example, the symptom–disorder relationship between endorsing a threshold number of drinks over 1 year and alcohol use disorder was significantly less specific for NLWs than other ethnoracial groups. NLWs were likelier to report this symptom despite not meeting the disorder criteria (Alegría & McGuire, 2003). NCLats and AfrCaribs may tend not to associate PLEs with special clinical significance due to these cultural interpretations.

### Study hypothesis

The clinical significance of PLEs varies complexly across ethnoracial groups, probably depending on the constellations of socio-structural and cultural factors that underlie the ethnoracial label. To clarify the resulting associations between ethnoracial labels and PLE clinical significance outcomes, we would need to unpack the relationship of PLEs to these underlying mechanisms. Our study hypothesis was rejected because it was overly simple, ignoring the multiplicity of factors that likely affect PLE prevalence and clinical significance.

### Clinical and nosological implications

Providers unfamiliar with cross-ethnoracial variation in PLE clinical significance may assign greater-than-warranted significance to PLEs in some ethnoracial groups (notably AsAms), potentially resulting in inappropriate diagnoses and interventions. Clinicians may also miss associations with specific mental health outcomes (e.g. CaribLats and hospitalization). In sum, using PLEs as markers of clinical severity (e.g. Kelleher et al., 2015) should be re-examined within ethnoracial groups, including their use to predict conversion to psychotic disorders (Zhang et al., 2019). To better calibrate diagnoses and treatment options, clinicians should obtain a thorough history of PLE characteristics, associated morbidities and impairments, barriers to care, patients' understandings of illness (e.g. spiritual causation), and current help-seeking expectations with instruments like the Cultural Formulation Interview (Lewis-Fernández et al., 2017).

Without biomarkers, psychiatric nosologies define diagnoses by identifying cutpoints in symptom dimensions that distinguish psychopathology from non-clinical distress (Maser et al., 2009). This process is made more complicated if the association of symptoms with morbidity and impairment can be affected by socio-structural factors, patients' patterns of expression, and clinicians' traditions of interpretation. This suggests the relationship between symptoms and disorders may not be constant across social groups (Alegría & McGuire, 2003; Kaiser et al., 2015).

Our findings challenge the conclusion that PLEs have uniform clinical significance across ethnoracial groups (e.g. DeVylder et al., 2014b). However, few studies have examined this issue directly, and the same limitations that impact the prevalence findings apply. Past studies deemphasized results suggestive of ethnoracial variation [e.g. in unemployment (DeVylder et al., 2014b), self-care (Oh et al., 2018)]; interpreted cross-sectional associations as indicating causation by PLEs rather than ethnoracial variation in PLE clinical significance (Vanheusden et al., 2008); utilized small sample sizes (Vanheusden et al., 2008); examined limited clinical significance outcomes (King et al., 2005); focused on subjective need for care (DeVylder et al., 2014a); or bundled diverse outcomes (Vanheusden et al., 2008). Instead, our study separately assessed mental healthcare use, morbidity, and impairment; excluded individuals whose clinical outcomes preceded PLE onset; used Cox regressions and adjustments for PLE duration to account for varying timeframes since PLE onset; stratified respondents by ethnoracial and Caribbean background; and compared across the major ethnoracial groups.

### Limitations

Our study has several limitations. First, the cross-sectional design prevented prospective examination of the temporal relationship between PLEs and adverse outcomes; we tackled this by excluding from the analysis outcomes that preceded PLE onset. However, we lack information on frequency of mental healthcare use, which may vary after PLE onset across ethnoracial groups, affecting associations with adverse outcomes. Second, we cannot examine the temporal sequence of CMD *v*. PLE first-onsets due to sample size limitations. Third, the associations between ethnoracial group and PLE clinical significance could be affected by our inclusion of individuals with clinician diagnoses of psychotic disorders, which varied across ethnoracial groups (6.2–17.5%). In NLAAS Latinos, this proportion (12.5%) was higher than the SCID-obtained clinical reappraisal of PLE-screener data, which showed that 7.0% of respondents with PLEs met DSM-IV criteria for psychotic disorder (Lewis-Fernández et al., 2009). Excluding the group with self-reported clinician diagnoses of psychotic disorder could have resulted in greater sampling bias than their inclusion. Fourth, the survey aimed to exclude via self-report PLEs related to substance use; however, this determination can be complex and vulnerable to misclassification. Fifth, survey non-response and selection bias, especially if differentially distributed across ethnoracial groups, may influence the strength of reported associations, despite weighting for non-response. Sixth, retrospective reports of lifetime experiences are subject to recall biases, especially concerning their temporal order. Seventh, due to the small sample size of respondents with PLEs, we could not further disaggregate the AsAm or Latino samples; we also had reduced statistical power to detect differences in less-common outcomes, such as suicide attempts. Finally, the age of the data may limit its applicability to the changing ethnoracial composition of the USA.

## Conclusions

Our analyses reveal less ethnoracial variation in PLE prevalence than previously described (Cohen & Marino, 2013; DeVylder et al., 2014a). AsAms reported significantly lower PLE prevalence than other U.S. ethnoracial groups, but the variation among Black and Caribbean-descent groups was inconsistent. Lower prevalence was not correlated with higher clinical significance. The clinical significance of PLEs varied across ethnoracial groups, being especially less salient among AsAms. Lack of familiarity with ethnoracial variation in PLE clinical significance may lead to the over-valuation of PLEs as markers of clinical severity in some ethnoracial groups. Future research could examine the association between PLEs, clinical severity, and conversion to psychotic disorder within ethnoracial groups, including the intersection of ethnoracial background with other social characteristics such as treatment barriers. The ethnoracial specificity of the relationships between other mental health symptoms and disorders could also be examined, as these relationships may vary across social groups.

## Supporting information

Lewis-Fernández et al. supplementary material Lewis-Fernández et al. supplementary material

## Supplementary material

The supplementary material for this article can be found at https://doi.org/10.1017/S0033291723001496.
