# Supplementary material for: Clinical significance of psychotic-like experiences across U.S. ethnoracial groups
Source: Psychol Med. 2023 Jun 5;53(16):7666–76. doi: 10.1017/S0033291723001496 (PMC10755236; doi:10.1017/S0033291723001496)
Supplement: Lewis-Fernández et al. supplementary material [file S0033291723001496sup001.docx]

**Supplementary Table 1. Clinical description of respondents with lifetime psychotic-like experiences in CPES, adjusted by age and gender (N=1,138).**

| **Number of individuals with PLEs** | **TOTAL** | **NCS-R Non-Latino Whites (I)** | **NLAAS Carib-Latinos (II)** | **NLAAS Non-Carib-Latinos (III)** | **NLAAS Asian Americans (IV)** | **NSAL African Americans (V)** | **NSAL Afro-Caribbeans (VI)** | **Omnibus Test** | **Benjamini- Hochberg post-hoc comparison** |
| --- | --- | --- | --- | --- | --- | --- | --- | --- | --- |
|  | **(n=1,138)** | **(n=181)^1,2^** | **(n=143)^2,3^** | **(n=136)^2,4^** | **(n=121)^2^** | **(n=427)^2^** | **(n=130)^2,5^** | **P Value** |  |
| **PLE characteristics** |  |  |  |  |  |  |  |  |  |
| Mean age of PLE onset (yrs) | 25.4 | 26.4 | 28.2 | 25.8 | 20.1 | 24.3 | 17.7 | 0.006 | I, II, V > VI |
| Number of PLEs |  |  | . | . | . | . | . | . |  |
| 1 | 73.5% | 77.7% | 58.3% | 69.3% | 76.5% | 71.3% | 68.9% | 0.006^6^ |  |
| 2 | 18.7% | 12.5% | 25.9% | 26.8% | 17.6% | 23.0% | 23.4% | . |  |
| 3+ | 7.8% | 9.8% | 15.8% | 3.8% | 5.9% | 5.7% | 7.7% | . |  |
| Any current PLEs | 30.7% | 28.6% | 41.6% | 31.3% | 24.5% | 32.2% | 47.5% | 0.33 | I < VI |
| **Type of lifetime PLEs** |  |  | . | . | . | . | . | . |  |
| Visual hallucinations | 69.2% | 68.3% | 66.5% | 64.3% | 68.3% | 73.5% | 89.7% | 0.24 |  |
| Auditory hallucinations | 43.3% | 40.2% | 57.0% | 49.2% | 39.3% | 44.3% | 24.0% | 0.16 |  |
| Thought insertion/withdrawal | 4.8% | 5.9% | 11.6% | 3.2% | 6.0% | 2.5% | 7.7% | 0.09 |  |
| Delusions of control | 2.2% | 1.2% | 8.0% | 2.1% | 2.4% | 2.9% | 5.2% | 0.10 | I < II |
| Delusions of reference | 8.1% | 9.1% | 12.1% | 9.1% | 8.7% | 4.9% | 7.4% | 0.37 |  |
| Persecutory delusions | 10.6% | 11.0% | 11.8% | 10.4% | 6.8% | 10.2% | 19.7% | 0.83 |  |

^1^Only the NCS-R random subsample that completed the psychosis symptom screener was included. ^2^Respondents with missing PLE data were dropped from the sample. ^3^Caribbean Latinos include respondents who identify as Puerto Rican, Cuban, or Dominican. ^4^Non-Caribbean Latinos include all other respondents who identify as Latinos (68.7% of Mexican origin). ^5^Afro-Caribbean respondents identified as Black and reported West Indian or Caribbean descent in their own, their parents’, or previous generations. ^6^None of the post-hoc tests remained significant after Benjamini-Hochberg correction.

Note: PLE: psychotic-like experiences.

**Supplementary Table 2. Demographic description of respondents with lifetime psychotic-like experiences in CPES (N=1,138).**

| **Number of individuals with PLEs** | **TOTAL** | **NCS-R Non-Latino Whites (I)** | **NLAAS Caribbean Latinos (II)** | **NLAAS Non-Caribbean Latinos (III)** | **NLAAS Asian Americans (IV)** | **NSAL African Americans (V)** | **NSAL Afro-Caribbeans (VI)** | **Omnibus Test** | **Benjamini- Hochberg post-hoc comparison** |
| --- | --- | --- | --- | --- | --- | --- | --- | --- | --- |
|  | **(n=1,138)** | **(n=181)^1,2^** | **(n=143)^2,3^** | **(n=136)^2,4^** | **(n=121)^2^** | **(n=427)^2^** | **(n=130)^2,5^** | **P Value** |  |
| **Demographic variables** |  |  |  |  |  |  |  |  |  |
| Migration Status |  |  |  |  |  |  |  |  |  |
| US-born | 83.0% | 95.5% | 40.4% | 54.5% | 33.2% | 98.4% | 51.0% | <0.0001 | II, III, IV, VI *v*. I |
| Foreign-born | 17.0% | 4.5% | 59.6% | 45.5% | 66.8% | 1.6% | 49.0% | . | II, III, IV, VI *v*. V |
| Age |  |  | . | . | . | . | . | . |  |
| 18-34 years | 37.3% | 31.8% | 44.3% | 46.3% | 49.3% | 36.6% | 51.8% | 0.0047^7^ |  |
| 35-49 years | 29.9% | 27.7% | 28.9% | 32.6% | 27.4% | 32.9% | 26.8% | . |  |
| 50+ years | 32.8% | 40.4% | 26.8% | 21.1% | 23.3% | 30.5% | 21.4% | . |  |
| Education |  |  | **.** | **.** | **.** | **.** | **.** | . |  |
| 11 years or less | 22.8% | 12.1% | 36.7% | 38.4% | 14.4% | 30.2% | 32.6% | <0.0001 | II, III, V *v.* I |
| 12 years | 29.0% | 32.2% | 24.0% | 22.4% | 11.3% | 32.3% | 26.9% | . | II, III, V, VI *v.* IV |
| 13-15 years | 29.5% | 31.2% | 28.7% | 26.6% | 38.5% | 27.0% | 24.3% | . |  |
| 16+ years | 18.7% | 24.4% | 10.6% | 12.6% | 35.8% | 10.6% | 16.3% | . |  |
| Gender |  |  | . | . | . | . | . | . |  |
| Male | 43.1% | 39.4% | 50.2% | 50.9% | 53.7% | 40.2% | 57.5% | 0.059 |  |
| Female | 56.9% | 60.6% | 49.8% | 49.1% | 46.3% | 59.8% | 42.5% | . |  |
| Household Income |  |  | . | . | . | . | . | . |  |
| $0-$14,999 | 27.5% | 21.3% | 35.0% | 33.3% | 18.4% | 35.2% | 31.1% | <0.0001 | I *v.* V |
| $15,000-$34,999 | 24.8% | 21.3% | 26.7% | 21.5% | 10.4% | 35.3% | 27.1% | . | II, III, V *v.* IV |
| $35,000-$74,999 | 30.1% | 34.9% | 22.6% | 30.1% | 26.3% | 23.4% | 36.8% | . |  |
| $75,000+ | 17.6% | 22.5% | 15.7% | 15.1% | 45.0% | 6.1% | 5.1% | . |  |
| Marital Status^6^ |  |  | . | . | . | . | . | . |  |
| Unmarried | 52.6% | 47.5% | 55.2% | 42.9% | 44.5% | 68.4% | 57.5% | <0.0001 | I, III, IV *v.* V |
| Married | 47.4% | 52.5% | 44.8% | 57.1% | 55.5% | 31.6% | 42.5% | . |  |

^1^Only the NCS-R random subsample that completed the psychosis symptom screener was included. ^2^Respondents with missing PLE data were dropped from the sample. ^3^Caribbean Latinos include respondents who identify as Puerto Rican, Cuban, or Dominican. ^4^Non-Caribbean Latinos include all other respondents who identify as Latinos (68.7% of Mexican origin). ^5^Afro-Caribbean respondents identified as Black and reported West Indian or Caribbean descent in their own, their parents’, or previous generations. ^6^Unmarried includes respondents who never married or who are widowed, separated, or divorced. ^7^None of the post-hoc tests remained significant after Benjamini-Hochberg correction

Note: PLE: psychotic-like experiences.

**Supplementary Table 3. Hazard ratios of lifetime, and odds ratios of current, mental health outcomes by lifetime psychotic-like experiences in CPES (Ns=914-1071), adjusted by nativity, other socio-demographic, and clinical variables.**

| **Lifetime Mental Health Outcomes** | | | | | | | | | | | | | | | | | | | | | | |  | |
| --- | --- | --- | --- | --- | --- | --- | --- | --- | --- | --- | --- | --- | --- | --- | --- | --- | --- | --- | --- | --- | --- | --- | --- | --- |
|  | **NCS-R Non-Latino Whites^1,2^** | | |  | **NLAAS Latinos^2,3^** | | | |  | | **NLAAS Asian Americans^2^** | | | | | |  | | **NSAL Black Americans^2,4^** | | |  | |  |
|  | HR | 95% CI | |  | HR | 95% CI | |  | | HR | | | 95% CI | | |  | | HR | | 95% CI | |  | |  |
|  | **(n=168)** | | |  | **(n=262)** | | |  | | **(n=118)** | | | | | |  | | **(n=523)** | | | |  | |  |
| **Psychiatric hospitalization after PLE onset (n=1071)** | 1 |  |  |  | 0.67 | 0.34 | 1.33 |  | | 0.52 | | 0.19 | | 1.44 | |  | | 0.67 | | 0.40 | 1.14 |  | |  |
|  | | | | | | | | | | | | | | | | | | | | | | | | |
|  | **(n=133)** | | |  | **(n=220)** | | |  | | **(n=108)** | | | | | |  | | **(n=453)** | | | |  | |  |
| **Outpatient mental health care after PLE onset (n=914)** | 1 |  |  |  | 0.68 | 0.35 | 1.33 |  | | 0.62 | | | 0.25 | | 1.54 |  | | **0.66** | | **0.44** | **0.99** | ** | |  |
|  | | | | | | | | | | | | | | | | | | | | | | | | |
|  | **(n=157)** | | |  | **(n=249)** | | |  | | **(n=110)** | | | | | |  | | **(n=496)** | | | |  | |  |
| **Suicidal ideation after PLE onset (n=1012)** | 1 |  |  |  | 0.65 | 0.35 | 1.20 |  | | 0.44 | | | 0.17 | | 1.13 |  | | 0.60 | | 0.34 | 1.03 |  | |  |
|  | | | | | | | | | | | | | | | | | | | | | | | | |
|  | **(n=167)** | | |  | **(n=249)** | | |  | | **(n=116)** | | | | | |  | | **(n=513)** | | | |  | |  |
| **Suicide attempt after PLE onset (n=1045)** | 1 |  |  |  | 0.48 | 0.17 | 1.35 |  | | 0.61 | | | 0.25 | | 1.51 |  | | 0.53 | | 0.22 | 1.27 |  | |  |
|  |  |  |  |  |  |  |  |  | |  | | |  | |  |  | |  | |  |  |  | |  |
| **Current Mental Health Outcomes** | | | | | | | | | | | | | | | | | | | | | | |  | |
|  | OR | 95% CI | |  | OR | 95% CI | |  | | OR | | | 95% CI | | |  | | OR | | 95% CI | |  | |  |
|  | **(n=82)** | | |  | **(n=279)** | | |  | | **(n=120)** | | | | | |  | | **(n=554)** | | | |  | |  |
| **Fair or poor mental health currently (n=1035)** | 1 |  |  |  | 0.59 | 0.29 | 1.18 |  | | 0.59 | | | 0.26 | | 1.36 |  | | 0.95 | | 0.47 | 1.92 |  | |  |
|  | | | | | | | | | | | | | | | | | | | | | | | | |
|  | **(n=179)** | | |  | **(n=279)** | | |  | | **(n=121)** | | | | | |  | | **(n=554)** | | | |  | |  |
| **Days out of role in last 30 days (n=1133)** | 1 |  |  |  | 0.50 | 0.18 | 1.43 |  | | 0.45 | | | 0.07 | | 3.00 |  | | **2.10** | | **1.12** | **3.93** | * | |  |

^1^Only the NCS-R random subsample that completed the psychosis symptom screener was included. ^2^Respondents with missing PLE data were dropped from the sample. ^3^Latinos combine Caribbean and non-Caribbean Latinos from the NLAAS. ^4^Black Americans combine African Americans and Blacks of Caribbean origin from the NSAL.

Note: Bolded values are statistically significant: *p<.05; **p<.01. Cox or logistic regressions adjusting for nativity, age of PLE onset, education, household annual income, marital status, and number of lifetime psychiatric disorders; logistic regressions also adjust for duration of PLEs. PLE: psychotic-like experiences.
